# Super hygroscopic nanofibrous membrane-based moisture pump for solar-driven indoor dehumidification

**DOI:** 10.1038/s41467-020-17118-3

**Published:** 2020-07-03

**Authors:** Yufei Zhang, Lei Wu, Xianfeng Wang, Jianyong Yu, Bin Ding

**Affiliations:** 1 State Key Laboratory for Modification of Chemical Fibers and Polymer Materials, College of Textiles, Donghua University, Shanghai, 201620 China; 2 College of Textile Materials and Engineering, Wuyi University, Jiangmen, 529020 China; 3 Innovation Center for Textile Science and Technology, Donghua University, Shanghai, 200051 China

**Keywords:** Nanoscale materials, Nanoscience and technology

## Abstract

Desiccants play vital roles in dehumidification and atmospheric water harvesting; however, current desiccants have mediocre hygroscopicity, limited recyclability, and high energy consumption. Herein, we report a wood-inspired moisture pump based on electrospun nanofibrous membrane for solar-driven continuous indoor dehumidification. The developed moisture pump with multilayer wood-like cellular networks and interconnected open channels is composed of a desiccant layer and a photothermal layer. The desiccant layer exhibits an unprecedented moisture absorption capacity of 3.01 g g^−1^ at 90% relative humidity (RH), fast moisture absorption and transport rates, enabling atmospheric water harvesting. The photothermal layer shows a high solar absorption of 93%, efficient solar thermal conversion, and good moisture permeability, thus promoting water evaporation. The moisture pump efficiently reduces the indoor relative humidity to a comfort level (40‒60% RH) under one-sun illumination. This work opens the way to develop new-generation, high-performance nanofibrous membrane-based desiccants for energy-efficient humidity control and atmospheric water harvesting.

## Abstract (web summary)

Desiccants are important for dehumidification, but application is hindered by limited hygroscopicity, recyclability, and energy efficiency. Here, the authors report a moisture pump comprised of an electrospun nanofibrous memebrane for solar-driven continuous indoor dehumidification.

## Introduction

Ambient humidity regulation has gained increasing concern in recent years due to its vital role in dehumidification^1,2^. One of the most critical challenges in indoor environments is regulating the humidity because high humidity can make people uncomfortable, cause furniture and clothes to become moldy, and accelerate damage to electronic equipment^3,4^. Thus, dehumidification is necessary in many high-humidity environments. The appropriate indoor relative humidity (RH) level for a healthy and comfortable environment in inhabited buildings ranges from 40 to 60%^5^. In addition, atmospheric humidity is widely considered as a redundant source of water, and energy is required to sustain comfortable humidity levels within living spaces^6^. Atmospheric water harvesting occurs during dehumidification, and the production of fresh water via moisture collection from humid air also shows great potential for sustainable water delivery^7–9^.

Conventional air-conditioning systems dehumidify air by cooling it below the dew point temperature to remove moisture via condensation, and then reheating it to the required temperature, which requires high energy consumption^10^. Various novel ideas for energy-saving technologies in buildings have recently emerged^11–13^. Therefore, independent humidity control systems need to be developed to achieve the desired balance between energy consumption and indoor comfort. As we all know, moisture can be autonomously transported from high-humidity to low-humidity environments through membranes. According to the thermo-osmosis theory, moisture can also be transported from a low-humidity to a high-humidity environment under the driving force of thermal gradients in a membrane^14^. This pathway or mode of moisture transport is referred to a moisture pump and is analogous to a heat pump. Extensive studies have recently been performed regarding the high-efficiency use of solar energy for surface-localized heating and steam generation^15–17^. Therefore, solar-driven moisture pump dehumidification technology is particularly attractive because of the abundance of solar energy, which results in significant energy-saving potential and ecological and economic benefits.

To regulate indoor humidity levels to achieve comfortable environments, an ideal desiccant material for a moisture pump should rapidly absorb moisture when the humidity level exceeds 60% RH. In addition, the desiccant must be highly hygroscopic, recyclable, have a fast moisture absorption rate, and be capable of driving the phase transition from gaseous water to liquid water. In this respect, metal–organic frameworks (MOFs) are attractive and promising due to their high-specific surface area and porosity, adjustable pore size, as well as a large number of hydrophilic active sites, thereby facilitating the rational design of the desired water sorption properties^18–22^. Yan et al. reported that the water vapor absorption capacity of MIL-101(Cr) reached 1.22 g g^−1^ at 25 °C and 90% RH, and its superior water vapor absorption made it a promising water vapor adsorbent^23^. Cao et al. proposed a silica gel-MIL-101(Cr)-based moisture-permeable panel prepared using a partial immersion method, which could reduce the indoor RH to a medium level^24^. However, the MIL-101(Cr) particles easily agglomerated, which reduced its water vapor transmission (WVT) and dehumidification capabilities. In addition, conventional membrane separation technique used for dehumidification is based on solution diffusion mechanism, which is energy-saving and environmentally friendly, but the moisture permeability is low^25^. To tackle the above issues, it is highly desirable to develop self-supporting and flexible MOF nanofibrous membrane (NFM)-based desiccants with highly porous structures and a large number of active sites for moisture absorption and water harvesting.

Electrospinning is a low-cost and scalable technology for preparing MOF NFMs because the nanofibrous structure of the resulting materials facilitates fast moisture absorption–desorption^26–28^. To further improve the moisture absorption sensitivity and capacity of NFM, a moisture-sensitive material such as LiCl can be loaded into/onto the nanofibers^28,29^. However, LiCl is prone to loss via deliquescence, and excessively high LiCl contents in composite desiccants will lead to problems such as salt precipitation and agglomeration, resulting in poor strength and recyclability^30–32^. To solve these issues, it is feasible to adopt dilute LiCl solutions to impregnate MOF NFMs. The hierarchical pore structures of both the MOF and nanofibers provide good support for LiCl to reduce the loss of LiCl, maintaining the moisture absorption stability of the NFM-based desiccant. Thus, the synergistic effect of the MOF and LiCl as well as the nanofibrous structure make it possible to design NFM-based desiccants with superior hygroscopicity, fast moisture absorption–desorption rates, and superior recyclability.

In trees, the microchannels of natural woods served as pathways to pump and transport water from the ground via transpiration^33,34^, which inspired the fabrication of biomimetic wood-like NFMs. Herein, we present a wood-inspired NFM-based moisture pump (biomimetic bilayer NFM) using a facile and scalable two-step electrospinning and impregnation method for solar-driven indoor dehumidification. To the best of our knowledge, there have been no previous reports on MOF NFM-based moisture pumps for continuous indoor dehumidification under sunlight illumination. The developed moisture pump with multilayer wood-like cellular networks and interconnected open channels is composed of a desiccant layer and a photothermal layer. The desiccant layer with high-specific surface area and porosity exhibits an unprecedented moisture absorption capacity of 3.01 g g^−1^ at 25 °C and 90% RH, fast moisture absorption and transport rates, as well as superior long-term stability, enabling atmospheric water harvesting. The photothermal layer displays a high solar absorption of 93%, efficient solar thermal conversion, and good moisture permeability, thus promoting water evaporation. This work reveals that a biomimetic NFM-based desiccant can potentially be applied for solar-driven moisture pump dehumidification and efficient atmospheric water harvesting.

## Results

### Wood-inspired design for the NFM-based moisture pump

**Fig. 1:**
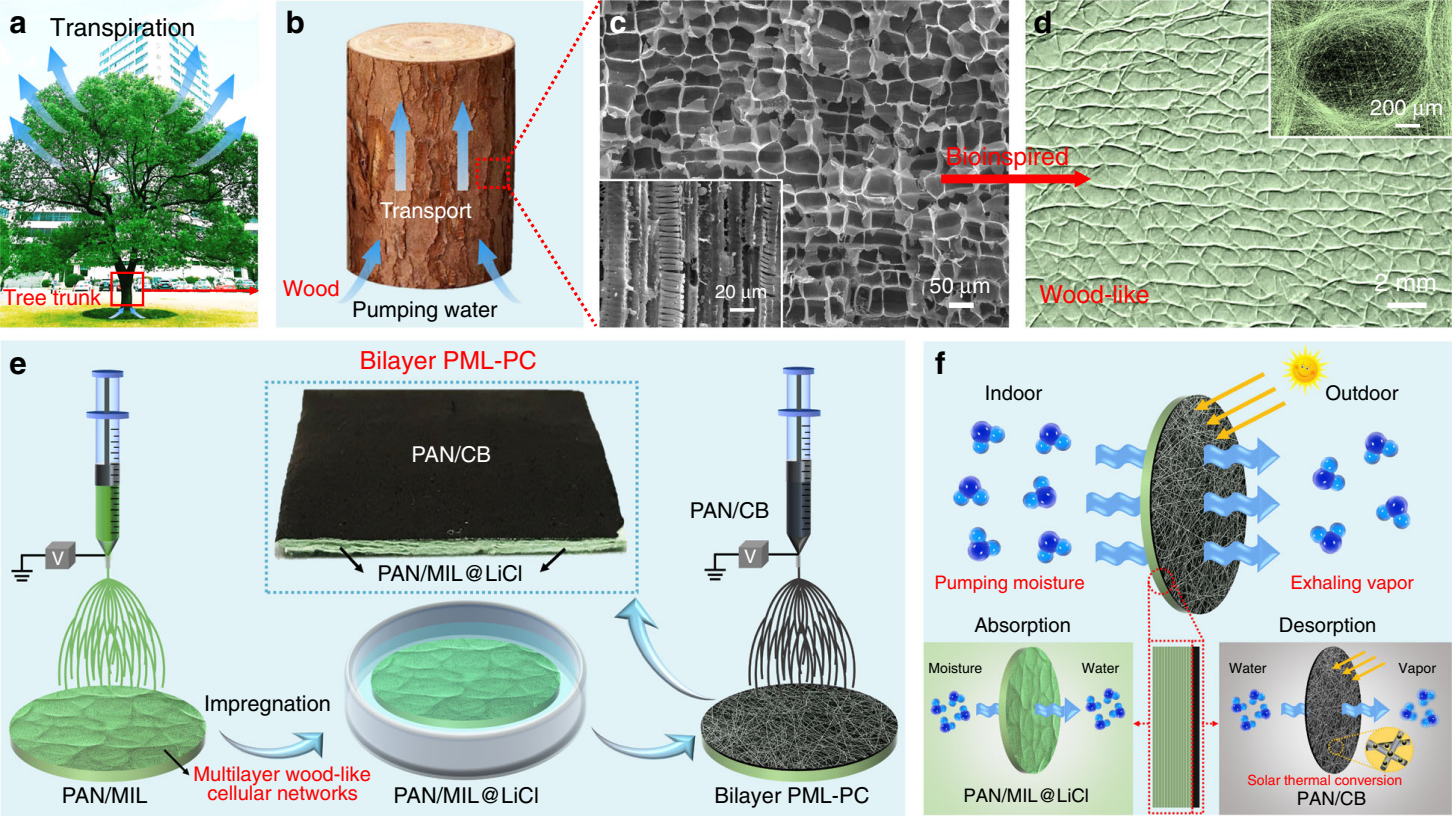
Wood-inspired design for the NFM-based moisture pump. **a**–**c** Natural wood obtained from a tree trunk contains special structures with horizontal hierarchical cellular networks and vertical interconnected channels that help pump and transport water up from the ground through transpiration. **d** The macro morphology of biomimetic PAN/MIL NFM with a wood-like cellular network structure. The inset shows a high-magnification SEM image of a single cell in the PAN/MIL NFM. **e** Schematic illustration of the fabrication of biomimetic bilayer PML-PC NFM. The PAN/CB nanofibers were directly electrospun on the multilayer wood-like cellular network substrate of the PAN/MIL@LiCl nanofibers. The middle inset shows the developed bilayer NFM. **f** Schematic structures of the moisture-permeable bilayer NFM and proposed moisture transport path under sunlight illumination. The absorbed water molecules are transported outdoors by passing through the desiccant layer and photothermal layer.

Natural wood from a tree trunk displays special structures with horizontal hierarchical cellular networks and vertical interconnected channels that pump and transport water from the ground upstream via transpiration (Fig. 1a–c). Inspired by this special structures and unique functional characteristics, a biomimetic wood-like NFM was developed (Fig. 1d) for moisture absorption and water vapor evaporation. The NFM surface presented a neatly arranged wood-like cellular network structure. High-magnification scanning electron microscopy (SEM) image showed a single cell with a pore size of ~1 mm (inset of Fig. 1d). As demonstrated in Fig. 1e, the fabrication began with the PAN/MIL-101(Cr) (PAN/MIL) NFM with a multilayer wood-like cellular network structure obtained directly by electrospinning. Then, the PAN/MIL NFM was impregnated by the LiCl solution to obtain PAN/MIL-101(Cr)@LiCl (PAN/MIL@LiCl) NFM. Due to the highly porous structure and fluffy multilayered architecture of PAN/MIL NFM, LiCl effectively penetrated the surface layer and entered the inner layer of the NFM and the porous nanofibers. Subsequently, the polyacrylonitrile/carbon black (PAN/CB) nanofibers were electrospun on the PAN/MIL@LiCl nanofibrous substrate to construct the biomimetic bilayer PAN/MIL@LiCl-PAN/CB (PML-PC) NFM (the middle inset of Fig. 1e). Schematic structures of the moisture-permeable bilayer NFM and proposed moisture transport path are shown in Fig. 1f. The biomimetic bilayer PML-PC NFM with wood-like cellular networks and interconnected open channels can perform moisture pumping and vapor exhaling. Notably, the desiccant layer (PAN/MIL@LiCl NFM) was responsible for absorbing moisture from indoor air, and absorbed water molecules were desorbed by the heat generated by solar thermal conversion of the photothermal layer (PAN/CB NFM) under sunlight illumination. The absorbed water molecules were transported outdoors by passing through the desiccant layer and photothermal layer. In this way, the proposed biomimetic bilayer NFM design can achieve high-efficiency and continuous indoor dehumidification under a solar irradiation, to serve as an NFM-based moisture pump.

### Morphology and structure characterizations of the desiccant layer

**Fig. 2:**
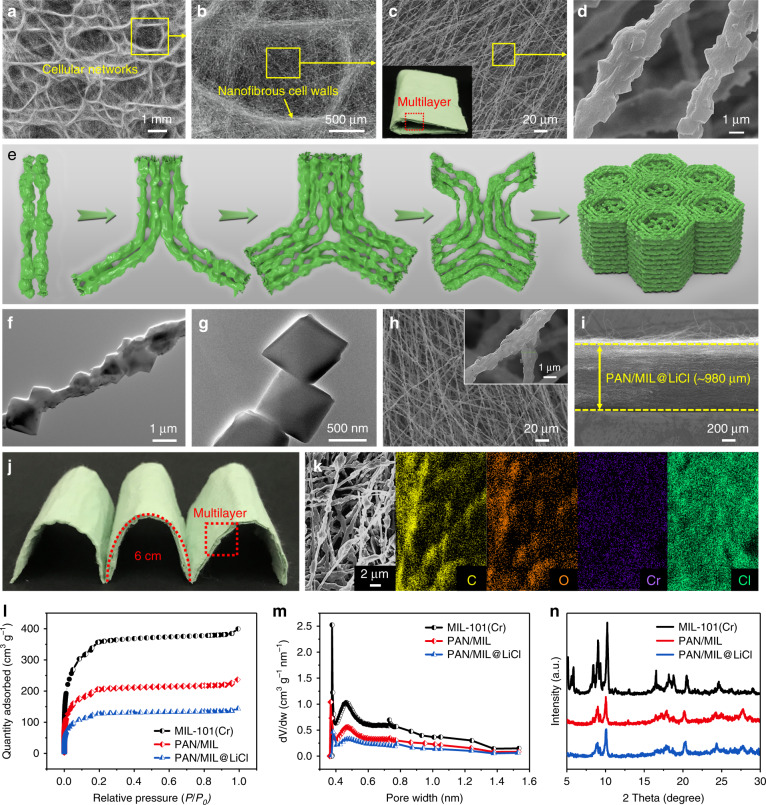
Morphology and structure characterizations of the desiccant layer. **a**–**d** SEM images at increasing magnifications demonstrating the biomimetic wood-like cellular network structure of PAN/MIL NFM. The inset in (**c**) is the as-prepared flexible PAN/MIL NFM photograph with a multilayered architecture. **e** Schematic showing the 3D self-assembly mechanism of PAN/MIL NFM with a multilayer wood-like cellular network structure. TEM images of (**f**) PAN/MIL nanofiber and (**g**) pure MIL-101(Cr) crystals. **h**, **i** Top-down and cross-section SEM images of PAN/MIL@LiCl NFM. The inset in (**h**) is a high-magnification SEM image. **j** Photograph demonstrating the flexibility of PAN/MIL@LiCl NFM and its multilayered architecture. **k** FE-SEM image and corresponding elemental maps of PAN/MIL@LiCl NFM. **l** N_2_ adsorption−desorption isotherms, **m** pore size distribution curves, and **n** XRD patterns of MIL-101(Cr) nanoparticles, PAN/MIL, and PAN/MIL@LiCl NFMs, respectively.

The micromorphologies of the biomimetic wood-like PAN/MIL NFM are shown in Fig. 2a–d. Of particular interest is that electrospinning technology enabled the assembly of PAN/MIL nanofibers into a highly ordered cellular network architecture consisting of unit cells, interconnected nanofibrous cell walls, and aligned and uniform nanofibers, mimicking the wood structure in both the horizontal and vertical directions. In addition, the merged nanofiber clusters were locally oriented along the edges of the cellular networks, which facilitated water diffusion and water evaporation. The as-prepared light green PAN/MIL NFM showed excellent flexibility and a multilayered architecture (inset of Fig. 2c). Typically, we were excited to find that MIL-101(Cr) nanoparticles were evenly distributed throughout nanofibers, showing that the developed nanofibers had a hierarchical roughness and nanoporous structure (Fig. 2d). The porous structure was conducive to moisture absorption and vapor diffusion^35^. The formation of a cellular network structure may be attributed to the competitive action of surface tension and electrostatic repulsion of the wet electrospun nanofibers^36,37^. We propose a simplified model to elucidate the three-dimensional (3D) self-assembly mechanism of the multilayer wood-like cellular network structure of PAN/MIL NFM (Fig. 2e). As the wet nanofibers were deposited and came into contact with the partially overlapped nanofiber clusters, surface tension may have driven the portion near the contact point to merge into nanofiber clusters, resulting in the charge accumulation on the surface of the nanofibers and the increase of electrostatic repulsion between the nanofibers. With the increase of electrostatic repulsion, the nanofibers far from the contact point may have bent outward. Finally, the nanofiber clusters were reversely bent to form a three-branched structure. Based on this, the deposited nanofibers were stacked layer-by-layer and formed 3D multilayer cellular networks composed of many three-branched clusters. Simultaneously, the addition of MIL-101(Cr) nanoparticles increased the conductivity of the electrospinning solution. The charged droplets acted as suspended clusters, which underwent rapid self-assembly via dissipation to minimize their energy^38^. Rapid stretching deformation due to the differential microelectric fields and additional solvent evaporation led to the formation of nanofiber assemblies with cellular networks.

Figure 2f presents the transmission electron microscopy (TEM) image of the PAN/MIL nanofiber, revealing that interconnected MIL-101(Cr) crystals were evenly arranged along the surface and inside the nanofibers. The dimension of single MIL-101(Cr) crystals was around 800 nm (Fig. 2g), indicating that the uniform nano-sized MIL-101(Cr) crystals and nanofibers had compatible sizes, which is helpful for electrospinning. PAN/MIL NFM was coated with LiCl via impregnation to improve its moisture absorption performance. The diameters of the PAN/MIL@LiCl nanofibers were uniform (Fig. 2h). High-magnification SEM image clearly exhibited that the nanofibers retained their hierarchical roughness and nanotextures (inset of Fig. 2h), showing that the LiCl coating did not substantially destroy the original morphology. The cross-sectional image of PAN/MIL@LiCl NFM in Fig. 2i shows that the fluffy multilayer NFM had a thickness of ~980 μm, and it also retained flexible nature and multilayered architecture (Fig. 2j). Interestingly, the multilayer NFM could be stripped layer-by-layer (Supplementary Fig. 1). Furthermore, the field emission scanning electron microscopy (FE-SEM) image and corresponding elemental maps of PAN/MIL@LiCl NFM were obtained (Fig. 2k). Cr and O derived from MIL-101(Cr) were uniformly distributed along the PAN nanofibers along with C, further revealing that the MIL-101(Cr) crystals were homogenously distributed on the nanofibers. In addition, the distribution of Cl confirmed that LiCl was completely and uniformly distributed throughout the membrane.

Considering that the porous structure feature is a vital element in desiccant materials, the samples were systematically investigated via N_2_ adsorption–desorption measurements. All isotherms exhibited typical type I isotherms without obvious hysteresis loops (Fig. 2l). The rapid adsorption equilibrium may be ascribed to micropore filling, indicating that a large amount of micropores existed in MIL-101(Cr) nanoparticles and NFMs. According to Horvath–Kawazoe (HK) model and density functional theory (DFT) method, the corresponding pore size distribution (PSD) curves showed that the micropore sizes were centered around 0.40–0.75 nm (Fig. 2m), which was larger than the kinetic diameter (0.27–0.32 nm) of a water molecule^39^, thus promoting moisture absorption and water vapor diffusion. The Brunauer–Emmett–Teller (BET)-specific surface area and pore structure parameters are listed in Supplementary Table 1. PAN/MIL NFM provided a large number of sites for LiCl loading due to its high-specific surface area (724 m^2^ g^−1^) and porosity. Nevertheless, the BET-specific surface area (*S*_BET_) decreased to 398 m^2^ g^−1^ after LiCl coating, implying that LiCl coating blocked some of the pores. As presented in Fig. 2n, characteristic peaks of MIL-101(Cr) were clearly visible in the X-ray diffraction (XRD) pattern of the synthesized MIL-101(Cr) crystals. The diffraction patterns of the PAN/MIL and PAN/MIL@LiCl NFMs closely matched that of MIL-101(Cr) crystals, revealing the existence of well-defined MOF in the NFMs and the amorphous structure of LiCl in PAN/MIL@LiCl NFM.

### Moisture absorption and water oozing behaviors of the desiccant layer

**Fig. 3:**
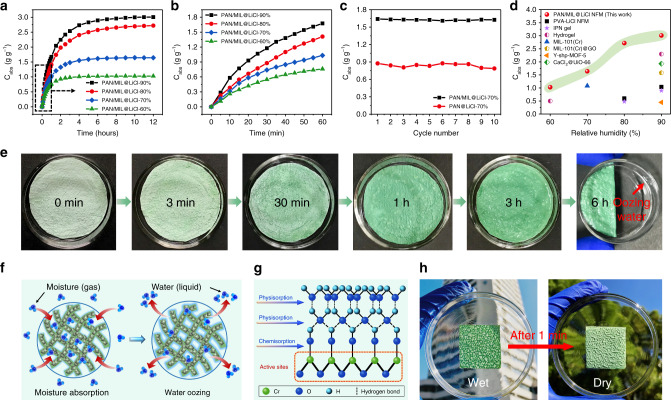
Moisture absorption and water oozing behaviors of the desiccant layer. **a**, **b** Moisture absorption kinetics of PAN/MIL@LiCl NFM at 25 °C and various humidities. **c** Cycling stability of moisture absorption−desorption of PAN/MIL@LiCl and PAN@LiCl NFMs at 25 °C and 70% RH (desorption at 100 °C). **d** A comparative chart of the moisture absorption capacity for some reported representative desiccant materials: PVA-LiCl NFM^27^, IPN gel^41^, and hydrogel^42^. MIL-101(Cr)^24^, MIL-101(Cr)@GO^23^, Y-shp-MOF-5^3^, and CaCl_2_@UiO-66^43^. **e** Optical photographs revealing surface moisture absorption, water oozing, and color change of PAN/MIL@LiCl NFM at 25 °C and 90% RH. **f** Schematic displaying the moisture absorption and water oozing behaviors of PAN/MIL@LiCl NFM. **g** Schematic showing the water absorption mechanism of MIL-101(Cr) in the PAN/MIL@LiCl NFM. **h** Optical photographs of the stripped monolayer PAN/MIL@LiCl NFM after moisture absorption and drying.

The moisture absorption behaviors of PAN/MIL@LiCl NFM were gravimetrically evaluated at 25 °C and various humidities. As observed in Fig. 3a, the moisture absorption capacities of dried PAN/MIL@LiCl NFM at 60%, 70%, 80%, and 90% RH reached 1.03, 1.64, 2.72, and 3.01 g g^−1^, respectively. It should be noted that the PAN/MIL@LiCl NFM quickly absorbed moisture from humid air within an hour (Fig. 3b). The absorbed water content and the time to reach saturation significantly increased with increasing ambient humidity because more water molecules were able to combine with active sites on the NFM. In terms of materials and structures, the synergistic effect of MIL-101(Cr) and LiCl in the desiccant layer imparted the material with super hygroscopicity, and the porous nanofibrous structure significantly increased the moisture absorption and transport rates. First, MIL-101 (Cr) had a large-specific surface area, high porosity, and rich hydrophilic active sites (Cr–O clusters), which were conducive to the absorption of water molecules. In addition, the nanofibrous structure facilitated fast moisture absorption, and LiCl reduced the water vapor pressure on the NFM surface, resulting in a greater driving force for water diffusion, thereby greatly improving the moisture absorption capacity and humidity sensitivity of PAN/MIL@LiCl NFM^40^.

To ensure low energy consumption in practical applications, continuous dehumidification is required for desiccants used in energy exchange systems, such as moisture pumps and dehumidifiers^41^. Thus, the moisture absorption−desorption cycling stability for the NFM must be considered. As shown in Fig. 3c, PAN@LiCl NFM was prepared (Supplementary methods) to provide a comparison to PAN/MIL@LiCl NFM. The moisture absorption capacity of PAN/MIL@LiCl NFM at 25 °C and 70% RH was much higher than that of PAN@LiCl NFM, indicating that the introduction of the MOF further improved the hygroscopicity and recyclability of the NFM. Evidently, the moisture absorption capacity of PAN/MIL NFM remained at 89.8% (0.79 g g^−1^) of its initial capacity (0.88 g g^−1^) after ten cycles, whereas PAN/MIL@LiCl NFM retained 99.4% (1.63 g g^−1^) of its initial capacity (1.64 g g^−1^). Significantly, the pore structure of MIL-101(Cr) and porous nanofibrous structure of PAN/MIL NFM provided good support for LiCl to form the PAN/MIL@LiCl NFM. With the assistance of the pores and channels of PAN/MIL@LiCl NFM, LiCl loss was avoided as much as possible, which enhanced the long-term stability of the NFM. To further evaluate the advantages of PAN/MIL@LiCl NFM for moisture absorption, the absorption capacity was quantitatively compared with previously reported desiccant materials (Fig. 3d)^3,23,24,28,41–43^. Obviously, PAN/MIL@LiCl NFM in this work exhibited superior hygroscopicity at 25 °C and various humidities compared with other desiccants. As a result, PAN/MIL@LiCl NFM can fully meet the needs of practical applications and can be easily operated at ambient temperature, surpassing the performance of other granular solid desiccant materials.

To observe water harvesting after moisture absorption, we placed PAN/MIL@LiCl NFM at 25 °C and 90% RH for a certain time. Figure 3e shows that the NFM quickly absorbed moisture in a short time (moisture absorption sensitivity), and the color of the NFM gradually deepened after moisture absorption. The NFM surface began to ooze water after 3 h, and the amount of liquid water increased with the moisture absorption time. Liquid water appeared in the watch glass after 6 h. Figure 3f displays the moisture absorption and water oozing behaviors of PAN/MIL@LiCl NFM. Water molecules were absorbed and liquefied on the surface of the NFM, mainly due to the presence of hygroscopic LiCl and MOF. Water then diffused into the porous networks of the NFM, enabling water to be captured from the moist air. The multilayer cellular networks of hydrophilic PAN/MIL@LiCl NFM facilitated water storage. As a result, liquid water was directly harvested from the humid air by absorbing moisture (gas) and oozing water using PAN/MIL@LiCl NFM without energy consumption (latent heat of evaporation and condensation)^41^. This unique property demonstrates the possibility of applying the PAN/MIL@LiCl NFM as an energy-efficient material that converts gaseous water into liquid water.

Figure 3g further elaborates on the water absorption mechanism of MIL-101(Cr) in PAN/MIL@LiCl NFM. First, water molecules were chemically absorbed on the active sites of the NFM, resulting in dissociation to form continuous hydroxyl groups. Subsequently, two adjacent hydroxyl groups absorbed water molecules via double hydrogen bonds to form the first physical absorption layer. At high RH, water molecules completely covered the NFM surface to form the first liquid water layer. Then, the reabsorbed water molecules formed a continuous network of liquid water layers by single hydrogen bonds^44–46^. The moisture absorption process was accompanied by irreversible (hysteretic) capillary condensation^7^. Eventually, the capacity of absorbed water molecules gradually reached saturation, and water oozing occurs. Although water oozing from the NFM still needs further research to improve efficiency, the use of NFM for moisture absorption and water harvesting is likely to become a promising system in the future. The phase transition of water molecules from the gas phase to the liquid phase using a dried membrane will open up a variety of possibilities for developing flexible desiccants for energy exchange systems with low energy consumption. In addition, it was particularly interesting that the stripped monolayer PAN/MIL@LiCl NFM was dried after 1 min under natural sunlight, demonstrating an obvious hierarchical cellular network structure (Fig. 3h). This indicated that the wood-like monolayer NFM exhibited an ultra-fast water diffusion and evaporation rate under sunlight illumination.

### Characterizations and properties of the photothermal layer

**Fig. 4:**
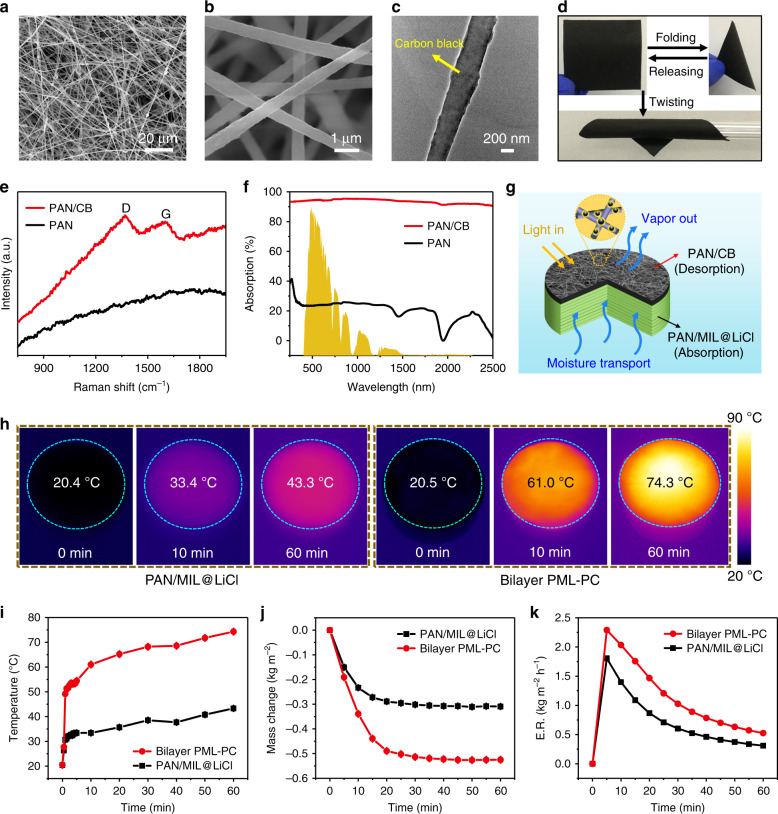
Characterizations and properties of the photothermal layer. **a**, **b** SEM images of PAN/CB NFM. **c** TEM image of PAN/CB nanofiber. **d** Photographs of folding and twisting tests of PAN/CB NFM. **e** Raman spectra of PAN/CB and PAN NFMs. **f** Light absorption of PAN/CB and PAN NFMs. Solar spectral irradiance (yellow shadow) weighted by standard AM1.5 G solar spectrum. **g** Schematic of the moisture transport and vapor-out processes of biomimetic bilayer PML-PC NFM. **h** IR thermal images revealing the surface temperature distribution of PAN/MIL@LiCl NFM and bilayer PML-PC NFM under one-sun illumination after a fixed time. **i** Surface temperature evolution of PAN/MIL@LiCl NFM and bilayer PML-PC NFM versus time under simulated sunlight irradiation of 1 kW m^−2^. **j** Mass change in the PAN/MIL@LiCl NFM and bilayer PML-PC NFM versus irradiation time under one-sun illumination. **k** Evolution of the water evaporation rate of PAN/MIL@LiCl NFM and bilayer PML-PC NFM as a function of time under a solar irradiation.

To achieve water vapor evaporation of the desiccant layer under sunlight, PAN/CB NFM was prepared as a photothermal layer based on PAN/MIL@LiCl NFM. The SEM images shown in Fig. 4a, b demonstrate that PAN/CB NFM was comprised of highly open 3D nanofiber networks (~480 nm) that provided channels for water evaporation. Figure 4c presents a TEM image of PAN/CB nanofibers that shows that CB nanoparticles were uniformly embedded in the nanofiber to form a rough surface. The rough surface of the PAN/CB nanofibers and the introduction of CB nanoparticles greatly enhanced the scattering of incident light within the photothermal layer, which improved the efficient absorption of broadband solar radiation. Thus, the generated heat was effectively confined within the photothermal layer with minimal loss to the air, which enhanced water evaporation. Photographs of folding and twisting PAN/CB NFM demonstrate its excellent flexibility and bending recovery (Fig. 4d). The Raman spectra also showed that CB nanoparticles were successfully incorporated into the PAN nanofibers (Fig. 4e). For the PAN NFM, there was no apparent characteristic peak, whereas two broad characteristic peaks near 1350 and 1600 cm^−1^ were observed in the Raman spectrum of PAN/CB NFM, which corresponded to the D and G bands of carbon, respectively^47,48^. PAN/CB NFM had a high solar absorption of 93% within a broad wavelength from 250 to 2500 nm in a standard solar spectrum (AM1.5 G), which was much higher than that of PAN NFM (Fig. 4f). To investigate the moisture permeability, WVT tests were carried out. The five curves of PAN, PAN/MIL, PAN/MIL@LiCl, PAN/CB, and bilayer PML-PC NFMs were nearly identical (Supplementary Fig. 2), indicating that the addition of MIL-101(Cr) or CB and the LiCl coating did not affect the WVT ability. This was ascribed to the nano-scale particle size and small aggregation domains of particles, which did not significantly change the pore and channel structures. In addition, numerous macropores were formed between the electrospun nanofibers. Therefore, both the desiccant layer and photothermal layer had good moisture permeability.

With a high broadband solar absorption, fast moisture transport, and good moisture permeability, the bilayer PML-PC NFM achieved efficient solar thermal conversion and enhanced water vapor evaporation under one-sun illumination. Figure 4g demonstrates the moisture transport and vapor-out processes of the biomimetic bilayer PML-PC NFM under a solar irradiation. To illustrate the importance of the photothermal layer, the surface temperature distribution of the PAN/MIL@LiCl NFM and bilayer PML-PC NFM under one-sun illumination was recorded by an infrared (IR) thermal camera (Fig. 4h). The initial surface temperatures of PAN/MIL@LiCl NFM (20.4 °C) and bilayer PML-PC NFM (20.5 °C) were nearly identical before illumination. However, the surface temperature of the bilayer PML-PC NFM rapidly increased within a few minutes and reached 74.3 °C after 60 min (Fig. 4i). The excellent heat confinement of the bilayer PML-PC NFM under low-intensity illumination shows that the material has efficient solar thermal conversion, which can promote solar-driven water evaporation. To investigate the evaporation performance, the cumulative mass losses of PAN/MIL@LiCl NFM and bilayer PML-PC NFM were measured under simulated sunlight irradiation of 1 kW m^−2^. The mass loss was nearly linear within the first 10 min, after which the evaporation rate slowed and gradually stabilized (Fig. 4j, k). Bilayer PML-PC NFM had a fast evaporation rate and achieved quick moisture desorption and dried out within 20 min under a solar irradiation. After 20 min of one-sun illumination, the evaporation rate of bilayer PML-PC NFM was 1.47 kg m^−2^ h^−1^, which was much higher than that of the NFM without a photothermal layer (0.87 kg m^−2^ h^−1^). Therefore, these facts confirm that the fast evaporation rate of bilayer PML-PC NFM under low irradiation was attributed to its superior moisture permeability, rapid moisture transport, and excellent light absorption, as well as its highly efficient light-to-heat conversion enabled by heat localization.

### Dehumidification performance of a moisture pump and application models

**Fig. 5:**
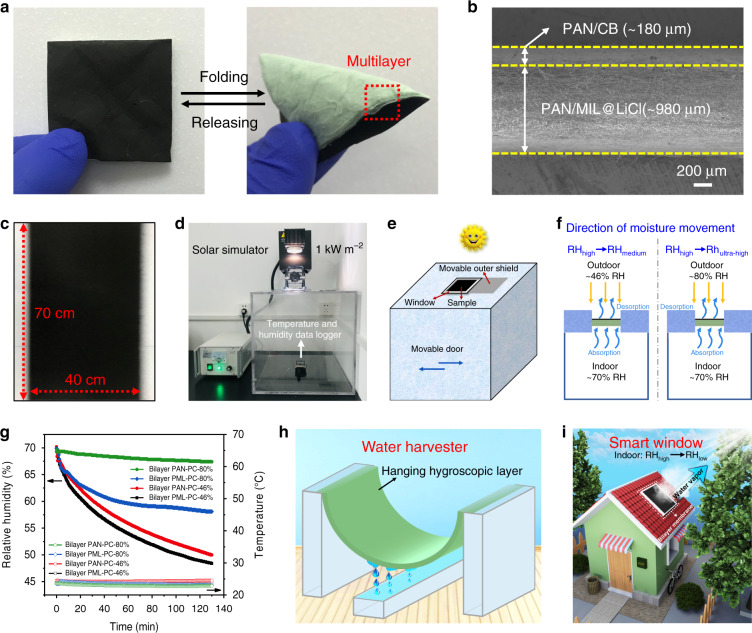
Dehumidification performance and application models. **a** Photographs of folding test of bilayer PML-PC NFM. **b** Cross-sectional SEM image of bilayer PML-PC NFM consisting of PAN/MIL@LiCl NFM and PAN/CB NFM. **c** Scalable demonstration of bilayer PML-PC NFM. **d** A scaled-down model of a house for continuous dehumidification testing. **e** Schematic illustration of the moisture pump model under simulated sunlight irradiation. When the light from the solar simulator illuminates the surface of bilayer PML-PC NFM, the moisture in the indoor air is pumped by bilayer PML-PC NFM and then evaporates in the form of water vapor. The blue arrows represent the direction of door movement. **f** Schematics displaying moisture movement through the moisture pump model to achieve indoor dehumidification. **g** Indoor relative humidity and temperature over time under one-sun illumination for bilayer PAN-PC and PML-PC NFMs when the device was placed in environments with ambient humidities of 46% and 80%, respectively. Plots showing the reduction in RH in a confined space as the bilayer NFMs absorb moisture from the humid air and evaporate outside. Application models of (**h**) an atmospheric water harvester by a novel hanging hygroscopic layer and (**i**) a smart window concept in which the bilayer membrane is used as an indoor dehumidification material.

To evaluate its practical applications, the prepared bilayer PML-PC NFM composed of a light green desiccant layer (PAN/MIL@LiCl NFM) and a black photothermal layer (PAN/CB NFM) was used as an NFM-based moisture pump. The bilayer PML-PC NFM had excellent flexibility and foldability (Fig. 5a), making it portable and convenient for practical environmental applications. In addition, the bilayer PML-PC NFM with a certain mechanical strength could be self-supported during application (Supplementary Fig. 3). Figure 5b shows the cross-section of bilayer PML-PC NFM consisting of PAN/MIL@LiCl NFM and PAN/CB NFM. PAN/CB NFM with a thickness of ~180 μm was covered on the fluffy multilayer PAN/MIL@LiCl NFM with a thickness of ~980 μm. It was worth mentioning that large area (70 cm × 40 cm) of bilayer PML-PC NFM could be readily fabricated using electrospinning (Fig. 5c), which is very important for practical applications.

A moisture pump prototype was constructed using the bilayer PML-PC NFM to evaluate its continuous dehumidification performance. The testing device consisted of a house model, a solar simulator, as well as a temperature and humidity data logger (Fig. 5d). The scaled-down model of a house was constructed with dimensions of 40 cm × 50 cm × 40 cm, and a window with an area of 6 cm × 6 cm was designed for bilayer membrane installation. Figure 5e depicts a schematic of the moisture pump model under simulated sunlight irradiation. When light from the solar simulator illuminated the surface of the bilayer PML-PC NFM, the moisture in the indoor air was pumped by bilayer PML-PC NFM and simultaneously transported outdoors in the form of water vapor. Figure 5f shows that the moisture actively moved from a high-humidity to a medium-humidity environment and also from a high-humidity to an ultra-high humidity environment through bilayer PML-PC NFM under sunlight irradiation. This is a unique advantage in terms of the dehumidification ability of the novel NFM-based moisture pump compared with traditional desiccants.

As a proof of concept, the RH reduction in a confined space was evaluated, in which the bilayer NFMs absorbed moisture from humid air and evaporated it outside (Fig. 5g). When the device was placed in an environment with an ambient humidity of 46%, the indoor RH was reduced from 70 to 48.9% within 2 h using bilayer PML-PC NFM, while it decreased to 50.7% when using bilayer PAN-PC NFM. This dehumidification process could be influenced by air convection. Therefore, to use the moisture pump for continuous dehumidification, the device was placed in an environment with an ambient humidity of 80%. The indoor RH in the house model steadily decreased from 70 to 58.3% within 2 h using bilayer PML-PC NFM, while it was only reduced to 67.5% using bilayer PAN-PC NFM. It was worth noting that the indoor temperature remained nearly constant, and only minimal temperature variations were caused by adsorption heat. These data confirmed that the dehumidification performance of the bilayer PML-PC NFM was significantly higher than that of the bilayer PAN-PC NFM, indicating that the synergistic effect of the MOF and LiCl played a vital role in the NFM-based moisture pump. Moreover, the final indoor humidity after dehumidification using the NFM-based moisture pump met the human body’s demand for comfortable environmental humidity (40–60% RH). Finally, the high-efficiency and continuous indoor dehumidification under one-sun illumination using bilayer PML-PC NFM was achieved, regardless of the external ambient humidity.

Based on the above-mentioned excellent properties of the as-prepared NFM, we look forward to the broad applications of such hygroscopic NFM and propose two application models. One of the applications is an atmospheric water harvester in which PAN/MIL@LiCl NFM is suspended in humid air for atmospheric water harvesting (Fig. 5h). Both sides of the NFM would be in contact with the humid air, which increases the capacity and rate of moisture absorption and water collection. The gaseous moisture in humid air can be successfully converted to liquid water, and water oozing from the surface of the hygroscopic NFM can drip from the bottom of the arc-shaped NFM into a water tank by gravity. The other model is a smart window concept (Fig. 5i) in which bilayer PML-PC NFM, which is capable of high-efficiency and continuous dehumidification under sunlight illumination, can potentially be used as a window screening membrane of a moisture-permeable window. Thus, it is expected to reduce the indoor humidity of living spaces suitable for human comfort.

## Discussion

In this study, the wood-inspired NFM-based moisture pump (biomimetic bilayer NFM) was designed using three criteria: (1) the bilayer NFM was composed of a desiccant layer (PAN/MIL@LiCl NFM) with a wood-like cellular network structure and a photothermal layer (PAN/CB NFM); (2) the desiccant layer must possess super hygroscopicity, fast moisture absorption and transport rates, as well as superior recyclability, and can even perform atmospheric water harvesting; (3) the bilayer NFM must be able to achieve high-efficiency and continuous indoor dehumidification while being directly powered by one-sun illumination. The first requirement is satisfied by directly electrospinning PAN/CB nanofibers onto a multilayer wood-like cellular network substrate of PAN/MIL@LiCl nanofibers. To satisfy the other two criteria, the moisture pump was designed to mimic plant transpiration. The synergistic effect of MIL-101(Cr) and LiCl in the desiccant layer imparted super hygroscopicity, and the nanofibrous structure significantly increased the moisture absorption–desorption rates. The LiCl coating and the fluffy multilayered architecture of the desiccant layer were beneficial to water harvesting, while the photothermal layer performed solar thermal conversion to generate heat for water vapor evaporation under sunlight illumination. The wood-like cellular networks and interconnected open channels of the biomimetic bilayer NFM are conducive to water transport and evaporation.

In summary, we have reported a wood-inspired NFM-based moisture pump using a facile and scalable two-step electrospinning and impregnation method for high-efficiency and continuous indoor dehumidification under sunlight illumination. The fluffy PAN/MIL@LiCl NFM with a multilayer wood-like cellular network structure possessed super hygroscopicity, a fast moisture absorption rate, as well as superior cyclability. Hydrophilic PAN/CB NFM with highly open 3D networks displayed a high broadband solar absorption of 93% and good moisture permeability and achieved efficient solar thermal conversion, which enhanced its water evaporation. As a result, the super hygroscopic desiccant layer exhibited, to the best of our knowledge, an unprecedented moisture absorption capacity of 3.01 g g^−1^ at 25 °C and 90% RH, achieving atmospheric water harvesting. The ability of the material to transform water from the gas phase to the liquid phase will open up a variety of possibilities for developing flexible desiccants for energy exchange systems with low energy consumption. The NFM-based moisture pump efficiently reduced the indoor RH to a moderate level under one-sun illumination to meet the human body’s comfort demands. To the best of our knowledge, this is the first report of a flexible electrospun NFM-based moisture pump. We envision that such a super hygroscopic NFM will provide new opportunities for developing atmospheric water harvesting, moisture-permeable windows on buildings, and humidity control inside electronic devices.

## Methods

### General

The experimental materials and detailed synthesis procedure of MIL-101(Cr) nanoparticles are given in the Supplementary methods.

### Preparation of wood-like PAN/MIL NFM

PAN/MIL NFM was fabricated by electrospinning technique. Briefly, 22 wt% of MIL-101(Cr) nanoparticles were added into DMF and then dispersed under ultrasonic treatment for 2 h. Subsequently, 8 wt% of PAN was added to the above solution with magnetic stirring. The blended solution was vigorously stirred for 12 h. PAN/MIL NFM was prepared through a DXES-3 electrospinning machine (Shanghai Oriental Flying Nanotechnology Co., Ltd, China). The PAN/MIL solution was loaded into plastic syringes, and the tip-roller distance of 15 cm was maintained. The feed rate of 1 mL h^−1^ and a stable voltage of 16 kV were applied, and the spinning process lasted 3 h. The ambient temperature and RH were kept at 23 ± 1 °C and 46 ± 3%, respectively. Finally, the as-prepared light green PAN/MIL NFM with a multilayer wood-like network structure was dried at 100 °C under vacuum.

### Preparation of PAN/MIL@LiCl NFM

The impregnation method was applied to coat LiCl into PAN/MIL NFM. The LiCl solution (concentration of 5 wt%) was prepared by adding LiCl particles to ethanol and stirring for 30 min. Subsequently, PAN/MIL NFM was impregnated in the LiCl solution for 30 min and then dried at 100 °C. The calculation of the LiCl coating ratio was detailed in Supporting information.

### Preparation of PAN/CB NFM

PAN/CB NFM was prepared by electrospinning technique. Briefly, 6 wt% of CB nanoparticles were added into DMF and then ultrasonic dispersed for 1 h. Subsequently, 10 wt% of PAN was added into the above solution. The blended solution was vigorously stirred for 12 h. Afterward, the electrospinning machine was applied, and the PAN/CB solution was transferred to five plastic syringes, and the tip-roller distance of 15 cm was maintained. The feed rate of 1 mL h^−1^ and a stable voltage of 20 kV were applied, and the spinning process lasted 6 h. The as-prepared PAN/CB NFM was deposited on a glossy paper covered roller. In addition, the ambient temperature and RH were 23 ± 1 °C and 46 ± 3%, respectively. Finally, the black PAN/CB NFM was dried at 100 °C under vacuum.

### Preparation of biomimetic bilayer PML-PC NFM

Preparation of the PAN/CB solution and regulation of spinning parameters of PAN/CB NFM as described above. Differently, biomimetic PAN/MIL@LiCl NFM was covered on the earthed metallic roller for the electrospinning of PAN/CB. After 6 h, the obtained bilayer NFM was dried at 100 °C for 2 h under vacuum. Finally, the prepared bilayer PAN/MIL@LiCl-PAN/CB NFM was denoted as bilayer PML-PC NFM.

### Characterization

The microstructures of the NFMs were characterized by SEM (VEGA 3), FE-SEM (S-4800), and TEM (JEM-2100). Optical images of the NFMs and the moisture absorption, water oozing processes were recorded by a digital video camera (Canon Powershot A1100IS). N_2_ adsorption–desorption isotherms were evaluated by physisorption analyzer (ASAP 2460), and the specific surface area and PSD were calculated using BET model, HK model, and DFT method, respectively. XRD patterns were determined by a D/Max-2550/PC (Rigaku Co., Japan) at Cu Kα radiation (*λ* = 1.5418 Å). The Raman spectra were recorded using a Raman spectrometer (inVia-Reflex) with an excitation laser of 532 nm. The reflectance and transmittance spectra were measured in the range of 250–2500 nm using a UV/Vis spectrophotometer (UV3600) equipped with integrating sphere. The thermal images and surface temperature distribution of the NFMs were recorded through an IR thermal camera (TiS75, Fluke, American). The WVT tests were investigated by the ASTM E96 positive cup standard using a WVT tester (YG 601H), and the measurements were carried out under 38 °C and 50% RH. Temperature and RH were recorded using a temperature and humidity data logger (Testo 175 H1). The tensile mechanical properties of the bilayer PML-PC NFM were characterized utilizing a tensile tester (XQ-1C). All samples were dried under vacuum at 100 °C for 3 h before testing.

### Moisture absorption measurement

The moisture absorption capacity, cycle stability, and water oozing performance were conducted using a constant temperature and humidity chamber. The WVT tester (YG 601H) was also used as a constant temperature and humidity chamber. The moisture absorption tests were performed at 25 °C and various humidities (60, 70, 80, and 90% RH). Before testing, the samples cut into circles with a radius of 3.4 cm were dried in an oven at 100 °C until their weight maintained unchanged, then placed in a constant temperature and humidity chamber. The samples should be taken out at a regular interval and weighed quickly in an electronic balance with an accuracy of 0.0001 g. During the absorption–desorption cyclic tests, the samples were maintained 6 h at 25 °C and 70% RH for moisture absorption and then kept 1 h at 100 °C for desorbing water vapor in every cycle. Water oozing after hygroscopic saturation was observed at 90% RH. The moisture absorption capacity of the sample is calculated by the following formula:

1 $$C_{abs} = \Delta m/m_0,$$

where, *C*_*abs*_ is the moisture absorption capacity based on unit weight of raw NFM (g g^−1^), Δ*m* is the moisture absorption quantity (g), and *m*_0_ is the weight of dried raw NFM (g).

### Solar evaporation experiment

The solar evaporation experiment was conducted in a room with constant temperature (~23 °C) and RH (~46%). Water vapor desorption testing was performed by a standard solar simulator (PLS-SXE300). The intensity of solar irradiation was controlled at 1 kW m^−2^. Prior to the solar evaporation measurement, the sample reached absorption saturation at 25 °C and 70% RH. The mass change of the sample over time under one-sun illumination was recorded every 5 min, which was used for calculating the water evaporation rates (kg m^−2^ h^−1^). Each sample was tested three times.

## Supplementary information

Supplementary Information

Peer Review File

## Data Availability

The authors declare that all data supporting the findings of this study are available within the article and its Supplementary information or from the corresponding author upon reasonable request. Source data are provided with this paper.

## Source data

Source Data
