# Supplementary material for: Super hygroscopic nanofibrous membrane-based moisture pump for solar-driven indoor dehumidification
Source: Nat Commun. 2020 Jul 3;11:3302. doi: 10.1038/s41467-020-17118-3 (PMC7335200; doi:10.1038/s41467-020-17118-3)
Supplement: Supplementary file 1 — Supplementary Information [file 41467_2020_17118_MOESM1_ESM.pdf]

Wood-Inspired Super Hygroscopic Nanofibrous Membrane-Based Moisture Pump for Solar-Driven Indoor Dehumidification

Zhang *et al.*

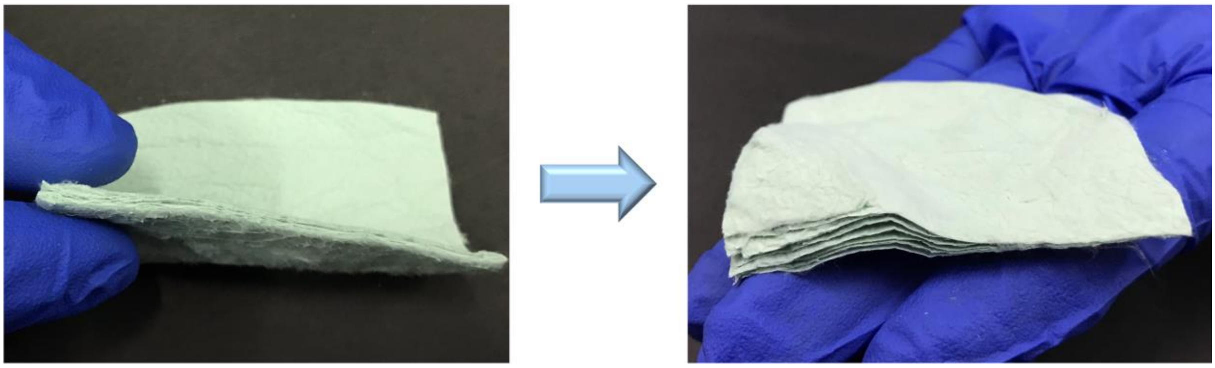

Supplementary Figure 1 The multilayer NFM is stripped.

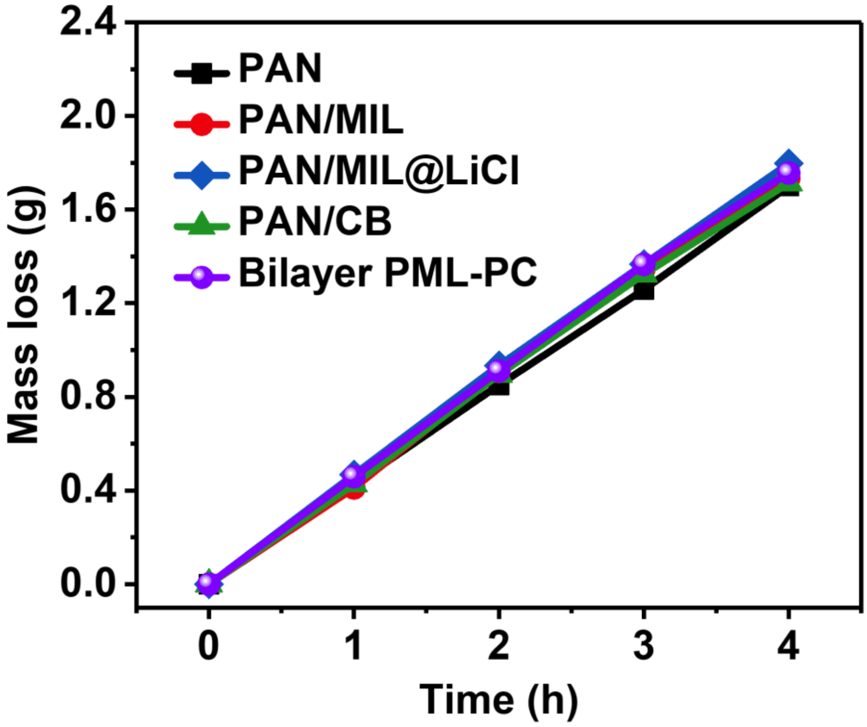

Supplementary Figure 2 Water vapor transmission test of PAN, PAN/MIL, PAN/MIL@LiCl, PAN/CB, and bilayer PML-PC NFMs.

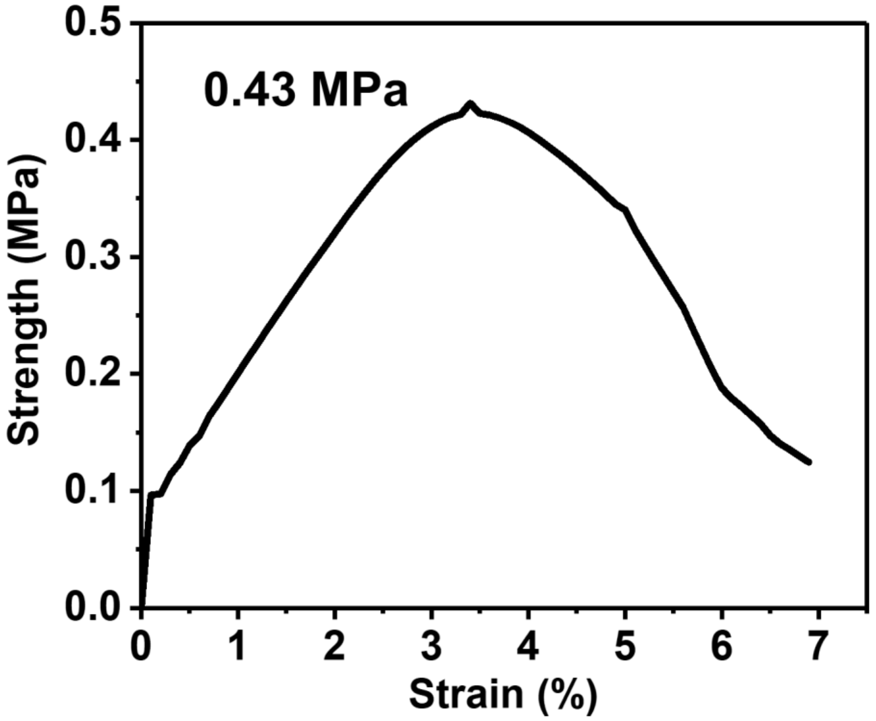

Supplementary Figure 3 Strength–strain curve of bilayer PML-PC NFM.

Supplementary Table 1 BET specific surface area and pore structure parameters of MIL-101(Cr) particles, PAN/MIL, and PAN/MIL@LiCl NFMs.

Sample	S_{BET}[a] (m^2 g^{-1})	V_{total}[b] (cm^3 g^{-1})	V_{micro}[c] (cm^3 g^{-1})	Mean pore width (nm)
MIL-101(Cr)	1276	0.5919	0.5122 (87)	0.7019
PAN/MIL	724	0.3472	0.2924 (84)	0.6928
PAN/MIL@LiCl	398	0.2125	0.1828 (86)	0.7021

Notes: [a]S_{BET} was calculated in the partial pressure (P/P_0) range of 0.01 to 0.1. [b]Total pore volume at relative pressure P/P_0 = 0.99. [c]Cumulative micropore volume with a diameter of ≤ 2 nm, the values in parentheses are the percentage of the micropore volume relative to the total pore volume.

Supplementary Note

The calculation of the LiCl coating ratio.

The LiCl coating ratio was obtained by the weight variation of NFM before and after impregnating LiCl, and then calculated according to the following formula:

$$\lambda = \frac{m - m_o}{S}$$

where, λ is the coating ratio based on the area of raw PAN/MIL NFM (g cm^{-2}), m_0 is the original weight of raw PAN/MIL NFM (g), m is the weight of dried PAN/MIL@LiCl NFM (g), and S refers to the area of raw PAN/MIL NFM (cm^2). The average LiCl coating ratio of PAN/MIL@LiCl NFM obtained by five experiments was listed in Supplementary Table 2.

Supplementary Table 2 The LiCl coating ratio of PAN/MIL@LiCl NFM.

Sample	λ (g cm^{-2})
PAN/MIL@LiCl	0.0103

Supplementary Methods

Materials. Chromium(III) nitrate nonahydrate ($Cr(NO_3)_3·9H_2O$, 99%), 1,4-benzene dicarboxylicacid (H_2BDC, 99%), absolute ethanol (EtOH, ≥ 99.7%), hydrofluoric acid (HF, ≥ 40%), and lithium chloride (LiCl, 98%) were obtained from Aladdin Biochemical Technology Co., Ltd., China. Carbon black (CB) nanoparticles were provided by Tianjin Zhengningxin Material Technology Co., Ltd., China. Polyacrylonitrile (PAN, M_w = 90 000) was purchased from Kaneka Co., Ltd., Japan. N,N-Dimethylfomamide (DMF, 99.5%) was supplied by Macklin Biochemical Co., Ltd., China. All chemicals were used without any further treatment. Deionized (DI) water was produced by a water purification system (UPT-11-20T).

Synthesis of MIL-101(Cr) nanoparticles. In a typical synthesis procedure, the reactant mixture with a molar composition of 1 $Cr(NO_3)_3·9H_2O$: 1 H_2BDC: 2.87 HF: 267 H_2O was poured into a Teflon-lined stainless steel autoclave and then followed by heating at 220 °C for 8 h. After being cooled to room temperature naturally, the green precipitates were obtained by centrifugation and washed several times with DI water, DMF, and hot ethanol. Finally, the product was dried in a vacuum oven at 150 °C for 12 h.

Preparation of PAN@LiCl NFM. The impregnation method was applied to coat LiCl into PAN NFM. The LiCl solution (concentration of 5 wt %) was prepared by adding LiCl particles to ethanol and stirring for 30 min. Subsequently, PAN NFM was impregnated in the LiCl solution for 30 min and then dried at 100 °C.
