# Supplementary material for: Super hygroscopic nanofibrous membrane-based moisture pump for solar-driven indoor dehumidification
Source: Nat Commun. 2020 Jul 3;11:3302. doi: 10.1038/s41467-020-17118-3 (PMC7335200; doi:10.1038/s41467-020-17118-3)
Supplement: Supplementary file 2 — Peer Review File [file 41467_2020_17118_MOESM2_ESM.docx]

Peer Review Information

**Journal:** Nature Communications

**Manuscript title:** Wood-Inspired Super Hygroscopic Nanofibrous Membrane-Based Moisture Pump for Solar-Driven Indoor Dehumidification

**Corresponding author name(s):** Prof Bin Ding

**Editorial notes:**

**none**

**Reviewer comments & decisions:**

**Reviewer comments, first version:**

Reviewer #1 (Remarks to the Author):

Following points need to be clarified in the revised manuscript before it is considered to be accepted.

1.The approach enables the naturally fragile MIL-101(Cr) crystals to convert into flexible PAN/MIL NFM with self-assembled 3D wood-like cellular networks and fluffy multilayered architecture using electrospinning technology directly. Are MOF particles easy to fall off from the fluffy NFM? Is the material stable during actual use? Furthermore, the developed moisture pump is composed of a desiccant layer and a photothermal layer. How are the two layers combined together? What about the binding force? How to control the binding force?

2. In Fig. 3e, the NFM quickly absorbed moisture in a short time (moisture absorption sensitivity). What does the moisture absorption sensitivity of PAN/MIL@LiCl NFM depend on? Is there a requirement for the humidity range of the environment? The authors need to explain about them. Furthermore, why did NFM release water molecules as a liquid state, not as a vapor state?

3. The authors compared moisture permeability of PAN, PAN/MIL, PAN/MIL@LiCl, and PAN/CB NFMs, but ignored the sample of bilayer PML-PC NFM. The moisture permeability of bilayer NFM is the vital data in this experiment. The authors need to supplement the experimental data.

4. Regarding the dehumidification performance under sunlight, the manuscript only exhibits experimental data under one-sun illumination, and simulated sunlight irradiation is 1 kW m-2. Whether increasing the intensity of sunlight irradiation can further improve the dehumidification rate of the material? Why does the manuscript not provide experimental data under different intensity of sunlight irradiation?


Reviewer #2 (Remarks to the Author):

Summary

The manuscript entitled “Wood-Inspired Super Hygroscopic Nanofibrous Membrane-Based Moisture Pump for Solar-Driven Indoor Dehumidification” by Zhang et al. shows the development of an effective wood-inspired moisture pump based on an electrospun nanofibrous membrane (NFM) for solar-driven continuous indoor dehumidification. The new developments allow Ding and his collaborators to fabricate a 2D fibrous multilayer device composed by a moisture pump and wood-like cellular networks vertical open channels. Moreover, the final material has been developed including both a desiccant layer and a photothermal layer in the same structure. The presented article contains a both basic experimental and application tests, which are vital to prove the claim with reliable data and in turn pave the way towards the application of this nanostructure in the field of smart windows and membranes.


General comments

In general, the work is accurate and well presented as a Nature Communications articles should be, moreover the presented results are certainly of interest to readers of this journal. The claims are supported by experimental evidences, moreover the material characterizations are very useful to prove the potential impact of the developed structures.
The article style is correct but it should be reviewed in a few points. Thus, I believe that the text needs some technical adjustments to be published. Therefore, I recommend that this manuscript can be published in Nature Communications after minor revision.


Specific comments

The article grammar, punctuation and style are quite adequate, but the manuscript need to be slightly proofread. The main problem is that Zhang et al. did not use articles in the right way, moreover there are a few mistakes in the use of singular/plural forms.


Going in details on the specific issues, here some comments are reported:


- Authors claim that PAN/MIL NFM shows multilayered architecture (with vertical pore interconnections), however it is not easy to appreciate this morphology in the images they reported. I suggest adjusting the claim (or the images) in the text.

- In the discussion of data reported in Figure 3a,b.c, authors claim that the synergistic effect of MIL and LiCl significantly improve the moisture absorption and transport rate of NMF but just data related to PAN/MIL@LiCl and PAN@LiCl are reported. I believe it would be better to provide more references in order to support the conclusions by previously published articles in that field.

- In the discussion of Figure 3f, authors claim that the wood-like monolayer NFM exhibits an ultra-fast water diffusion and evaporation rate. However, no quantitative data connected to this phenomenon are reported here. Please modify the text to fit the discussion with the image content.

- The schematic reported in Figure 5e is not very clear. Red arrows are pointing to different directions and no explanation is written nor in the figure nor in the caption. Legend for arrows is not reported. Please improve the schematic to make more clear the pump model the authors proposed.

- Figures 4i 4j and 4h report interesting quantitative data that are very useful to prove the final applicability of the developed materials. Anyway, the bars representing the standard deviations should be included. Moreover, all the necessary pieces of information connected with the statistical treatment of data (e.g. number of experiments performed per each condition) should be included.

- There is a mistake in the section titles. “Results” should be replaced by “Results and Discussion” while “Discussion” should be replaced by “Conclusions”.


Conclusion

The topic of this manuscript falls within the scope of Nature Communications. I like the concept and material development/characterization proposed in this paper, moreover the manuscript includes in-depth studies to support the author claim from both fundamental and application point of views, anyway I think the manuscript needs a few improvements. I believe the article is of sufficient quality and novelty to meet the Nature Communications publication standard after a minor revision.


Reviewer #3 (Remarks to the Author):

The manuscript by Zhang et al reports on the preparation of a polyacrylonitrile(PAN)-based composite membrane for sun-light driven dehumidification. Key of the finding reported here the combination of a bilayer system with a layer of PAN / carbon black layer for sunlight collection and another layer with PAN / metal organic framework (MOF)-LiCl nanoparticles for the conversion of gaseous water to liquid water. Once the liquid water has been collected the energy collected from sunlight will evaporate this water to outside and thereby dehumidify a room. Interesting the authors report on a special morphology of the electrospun membranes which they claim as wood-inspired morphology and is surely special for electrospun membranes. According to the authors this special morphology helps in dehumidification.
The manuscript is surely of interest for the readers of Nature Communication and could be also very inspiring for future work. The manuscript is well written and presents tons of experimental details. Only the figures are too small and the labels on many figures might be hard to read, which has to be improved.
Of course dehumidification by the MOF used here (MIL-101(Cr) and the concept of using membranes for dehumidification is not new – please compare to doi: 10.1016/j.isci.2019.05.013 Membranes2015, 5, 722-738; doi:10.3390/membranes5040722, https://doi.org/10.1002/admi.201901310. The authors should compare their systems with existing systems quantitatively and thereby elaborate the novelty of their system.
Here, the inspiration by wood morphology is somewhat exaggerated. The explanation for the cellular-like structure of the membranes, which unique for electrospinning indeed, are assumptions and should be also identified accordingly (see page 10, top paragraph. Surely, this morphology was obtained accidentally and not planned. Therefore, one should not claim wood-inspired but better wood-like. In this context how it the scale of the wood morphology. Does it match the morphology of the present membranes?
Further, is this morphology unique for composites with PAN/MOF or could it be obtained by other polymers or MOFs as well?
In Fig. 5 the authors suggest real applications which are of course of interest. Here, the question comes up about the UV stability of the sunlight-collecting layer – will it become brittle after some time? Has this been quantified?
As a minor remark: why the photo in Fig. 3h was taken with different backgrounds?

In conclusion, the manuscript by Zhang et al. is a very interesting manuscript and could be published in Nature Communication after minor revision.

**Author rebuttal, first version:**

***Response to Reviewer 1***

**Comments to the Author:**

Following points need to be clarified in the revised manuscript before it is considered to be accepted.

1. The approach enables the naturally fragile MIL-101(Cr) crystals to convert into flexible PAN/MIL NFM with self-assembled 3D wood-like cellular networks and fluffy multilayered architecture using electrospinning technology directly. Are MOF particles easy to fall off from the fluffy NFM? Is the material stable during actual use? Furthermore, the developed moisture pump is composed of a desiccant layer and a photothermal layer. How are the two layers combined together? What about the binding force? How to control the binding force?

***Response:*** *Thank you for your worthy questions. MOF particles did not fall off from the fluffy NFM. Electrospinning was able to convert MOF particles into self-supported and flexible MOF NFM (inset of Fig. 2c). Additionally, the nano-sized MOF particles and electrospun nanofibers had a good size suitability, and further decreased their agglomeration possibility. As shown in Fig. 2d and 2h, MIL-101(Cr) nanoparticles were evenly and firmly distributed throughout nanofibers. Furthermore, Fig. 2f presents the TEM image of the PAN/MIL nanofiber, revealing that interconnected MIL-101(Cr) crystals were evenly arranged along the surface and inside the nanofiber. SEM and TEM images illustrated the particles penetrated or embedded in the nanofibers. Thus, the material is stable during actual use.*

*The bilayer NFM is composed of a desiccant layer (PAN/MIL@LiCl NFM)* *and a photothermal layer (PAN/CB NFM). As demonstrated in Fig. 1e, the fabrication began with the PAN/MIL NFM obtained directly by electrospinning. Then, the PAN/MIL NFM was impregnated by the LiCl solution to obtain PAN/MIL@LiCl NFM. Subsequently, the PAN/CB nanofibers were electrospun on the prepared PAN/MIL@LiCl nanofibrous substrate to construct the biomimetic bilayer NFM (the middle inset of Fig. 1e). The binding force between the two layers is strong enough so that it will not slip or separate even after being folded (Fig. 5a). During electrospinning, the solvent-containing (wet) state of the polymer can be maintained by controlling the evaporation rate of the solvent to increase adhesion between the polymer fibers. In addition, the binding force between interfaces can be enhanced by adding adhesive crosslinking agent in the future work.*

2. In Fig. 3e, the NFM quickly absorbed moisture in a short time (moisture absorption sensitivity). What does the moisture absorption sensitivity of PAN/MIL@LiCl NFM depend on? Is there a requirement for the humidity range of the environment? The authors need to explain about them. Furthermore, why did NFM release water molecules as a liquid state, not as a vapor state?

***Response:*** *Thank you very much for your careful review and valuable suggestion. The synergistic effect of MIL-101(Cr) and LiCl in the desiccant layer imparted the material with moisture absorption sensitivity. MIL-101 (Cr) had a large specific surface area, high porosity, and rich hydrophilic active sites (Cr-O clusters), which were conducive to the absorption of water molecules. In addition, the nanofibrous structure facilitated fast moisture absorption, and LiCl reduced the water vapor pressure on the NFM surface, resulting in a greater driving force for water diffusion, thereby greatly improving the moisture absorption capacity and humidity sensitivity of the PAN/MIL@LiCl NFM. The ambient humidity of the experiment was set at 60%, 70%, 80%, and 90% RH, respectively. When the ambient humidity was higher than 60%, the PAN/MIL@LiCl NFM had excellent moisture absorption sensitivity. The PAN/MIL@LiCl NFM quickly absorbed moisture from humid air within an hour (Fig. 3b). The absorbed water content and the time to reach saturation significantly increased with increasing ambient humidity because more water molecules were able to combine with active sites on the NFM. To regulate indoor humidity levels to achieve a comfortable environment, an ideal desiccant material for a moisture pump should rapidly absorb moisture when the humidity level exceeds 60% RH.*

*NFM released water molecules as a liquid state, not as a vapor state. Water molecules were absorbed and liquefied on the surface of NFM, mainly due to the hygroscopic LiCl and MOF. Fig. 3g elaborates on the water absorption mechanism of MIL-101(Cr) in PAN/MIL@LiCl NFM. First, water molecules were chemically absorbed on the active sites of the NFM, resulting in dissociation to form continuous hydroxyl groups. Subsequently, two adjacent hydroxyl groups were able to absorb water molecules by double hydrogen bonds to form the first physical absorption layer. At high RH, water molecules completely covered the NFM surface to form the first liquid water layer. Then, the reabsorbed water molecules formed a continuous network of liquid water layers by single hydrogen bonds. The moisture absorption process was accompanied by irreversible (hysteretic) capillary condensation. Eventually, the capacity of absorbed water molecules gradually reached saturation, and water oozing occurs.* *This unique property demonstrates the possibility of applying the PAN/MIL@LiCl NFM as an energy-efficient material that converts gaseous water into liquid water.*

3. The authors compared moisture permeability of PAN, PAN/MIL, PAN/MIL@LiCl, and PAN/CB NFMs, but ignored the sample of bilayer PML-PC NFM. The moisture permeability of bilayer NFM is the vital data in this experiment. The authors need to supplement the experimental data.

***Response:*** *Thank you for your valuable suggestion. The moisture permeability of bilayer NFM has been supplemented in Supplementary Information (Kindly see Supplementary Figure 2). We have added the relevant information in the revised manuscript (Please see pages 19-20 lines 356-358).*

***Supplementary Figure 2*** *Water vapor transmission test of PAN, PAN/MIL, PAN/MIL@LiCl, PAN/CB, and bilayer PML-PC NFMs.*

4. Regarding the dehumidification performance under sunlight, the manuscript only exhibits experimental data under one-sun illumination, and simulated sunlight irradiation is 1 kW m^-2^. Whether increasing the intensity of sunlight irradiation can further improve the dehumidification rate of the material? Why does the manuscript not provide experimental data under different intensity of sunlight irradiation?

***Response:*** *We truly appreciate your valuable questions. Increasing the intensity of sunlight irradiation can further improve the dehumidification rate of the material. When the light from the solar simulator illuminated the surface of bilayer PML-PC NFM, the moisture in the indoor air was pumped by bilayer PML-PC NFM and simultaneously transported outdoors in the form of water vapor. As the intensity of sunlight increases, the temperature of the NFM surface increases, which promotes the evaporation of water vapor. However, considering the actual application environment of the material, simulated sunlight irradiation of 1 kW m^−2^ matches the actual light intensity. Furthermore, under one-sun illumination, the NFM-based moisture pump can efficiently reduce the indoor RH to a medium level to meet the human body’s comfort demands. Therefore, experimental data under different intensity of sunlight irradiation is not necessary in this manuscript.*

***Response to Reviewer 2***

**Comments to the Author:**

**Summary:** The manuscript entitled “Wood-Inspired Super Hygroscopic Nanofibrous Membrane-Based Moisture Pump for Solar-Driven Indoor Dehumidification” by Zhang et al. shows the development of an effective wood-inspired moisture pump based on an electrospun nanofibrous membrane (NFM) for solar-driven continuous indoor dehumidification. The new developments allow Ding and his collaborators to fabricate a 2D fibrous multilayer device composed by a moisture pump and wood-like cellular networks vertical open channels. Moreover, the final material has been developed including both a desiccant layer and a photothermal layer in the same structure. The presented article contains a both basic experimental and application tests, which are vital to prove the claim with reliable data and in turn pave the way towards the application of this nanostructure in the field of smart windows and membranes.

**General comments:** In general, the work is accurate and well presented as a Nature Communications articles should be, moreover the presented results are certainly of interest to readers of this journal. The claims are supported by experimental evidences, moreover the material characterizations are very useful to prove the potential impact of the developed structures.

The article style is correct but it should be reviewed in a few points. Thus, I believe that the text needs some technical adjustments to be published. Therefore, I recommend that this manuscript can be published in Nature Communications after minor revision.

***Response:*** *Thank you very much for your positive comments. We highly appreciate the valuable suggestions. Comments have been addressed one-by-one as follows.*

**Specific comments:** The article grammar, punctuation and style are quite adequate, but the manuscript need to be slightly proofread. The main problem is that Zhang et al. did not use articles in the right way, moreover there are a few mistakes in the use of singular/plural forms.

***Response:*** *Thanks for pointing these mistakes out. We have carefully checked the manuscript and corrected the mistakes accordingly.*

Going in details on the specific issues, here some comments are reported:

1. Authors claim that PAN/MIL NFM shows multilayered architecture (with vertical pore interconnections), however it is not easy to appreciate this morphology in the images they reported. I suggest adjusting the claim (or the images) in the text.

***Response:*** *Thank you very much for your valuable comment. According to your kind suggestion, we have adjusted the claim “vertical open channels” to “interconnected open channels” in the revised manuscript (Please see page 2 line 21; page 6 line 103; page 7 line 134; page 28 line 501).*

2. In the discussion of data reported in Figure 3a, b, c, authors claim that the synergistic effect of MIL and LiCl significantly improve the moisture absorption and transport rate of NMF but just data related to PAN/MIL@LiCl and PAN@LiCl are reported. I believe it would be better to provide more references in order to support the conclusions by previously published articles in that field.

***Response:*** *We truly appreciate your valuable suggestion. “In terms of materials and structures, the synergistic effect of MIL-101(Cr) and LiCl in the desiccant layer imparted the material with super hygroscopicity, and the porous nanofibrous structure significantly increased the moisture absorption and transport rates.” In addition to the experimental data (Fig. 3a-c) provided in the manuscript, the relevant references (ref. 3, 23, 24, 27, 28, 39, 40-42) have been cited in the introduction and Fig. 3d, and the relevant statement has been provided in the revised manuscript (see page 4 lines 71-77; page 5 lines 83-85; page 14 lines 258-262; page 15 lines 278-280). Thus, the synergistic effect of MOF and LiCl as well as the nanofibrous structure make it possible to design NFM-based desiccants with super hygroscopicity, fast moisture absorption–desorption rates, and superior recyclability.*

3. In the discussion of Figure 3h, authors claim that the wood-like monolayer NFM exhibits an ultra-fast water diffusion and evaporation rate. However, no quantitative data connected to this phenomenon are reported here. Please modify the text to fit the discussion with the image content.

***Response:*** *We are very grateful for your valuable comment.* *According to your kind suggestion, we have modified the caption of Fig. 3h in the revised manuscript (page 18 lines 333-334) to fit the discussion with the image content. Actually, the purpose of Fig. 3h is to show the surface morphology of the PAN/MIL@LiCl NFM after moisture absorption and drying, while the optical photographs of the stripped monolayer PAN/MIL@LiCl NFM exhibited a more obvious hierarchical cellular network structure. The quick-dry performance here is an accompanying performance demonstration. Mass change of the stripped monolayer PAN/MIL@LiCl NFM is not tested, but the statement “NFM was dried after 1 min under natural sunlight” is actually measured. In addition, the quick-dry data of the stripped monolayer PAN/MIL@LiCl NFM is unnecessary in this manuscript, because the water evaporation rate of the PAN/MIL@LiCl NFM and bilayer PML-PC NFM is the focus of this study, the corresponding data has been obtained (Fig. 4j and k) and the relevant analysis has been performed (see page 21 lines 378-389). Compared with the data in Fig. 4k, the wood-like monolayer NFM exhibited an ultra-fast water diffusion and evaporation rate under sunlight illumination, thus the statement (page 17 lines 316-320) is consistent.*

4. The schematic reported in Figure 5e is not very clear. Red arrows are pointing to different directions and no explanation is written nor in the figure nor in the caption. Legend for arrows is not reported. Please improve the schematic to make more clear the pump model the authors proposed.

***Response:*** *Thank you very much for your careful review. The red arrows in Fig. 5e do not represent the direction,* *the blue arrows represent the direction of door movement. We have made adjustments to the red arrows in Fig. 5e and added explanation to the blue arrows in the caption (see page 27 line 474).*

5. Figures 4i 4j and 4h report interesting quantitative data that are very useful to prove the final applicability of the developed materials. Anyway, the bars representing the standard deviations should be included. Moreover, all the necessary pieces of information connected with the statistical treatment of data (e.g. number of experiments performed per each condition) should be included.

***Response:*** *Thank you for your valuable comment and thoughtful suggestion. We have redrawn Fig. 4i and 4j in the revised manuscript, including the bars representing the standard deviations. Moreover, we have also provided the raw data of three experimental tests for each sample in “Source Data”. The value of the surface temperature distribution in Fig. 4h is the average value of three experimental tests. In addition, the water evaporation rate in Fig. 4k was calculated according to the average of mass change in Fig. 4j. We have added the relevant information about the number of experiments performed per each condition in the revised manuscript (Please see page 33 lines 603-604).*

***Fig. 4 h*** *IR thermal images revealing the surface temperature distribution of PAN/MIL@LiCl NFM and bilayer PML-PC NFM under one-sun illumination after a fixed time.* ***i*** *Surface temperature evolution of PAN/MIL@LiCl NFM and bilayer PML-PC NFM versus time under simulated sunlight irradiation of 1 kW m^-2^.* ***j*** *Mass change in the PAN/MIL@LiCl NFM and bilayer PML-PC NFM versus irradiation time under one-sun illumination.* ***k*** *Evolution of the water evaporation rate of PAN/MIL@LiCl NFM and bilayer PML-PC NFM as a function of time under a solar irradiation.*

6. There is a mistake in the section titles. “Results” should be replaced by “Results and Discussion” while “Discussion” should be replaced by “Conclusions”.

***Response:*** *Thank you very much for the suggestion. According to the format requirements given by the editor, “Results” is separate from “Discussion”, so the two sections are titled “Results” and “Discussion”, respectively.*

**Conclusion:** The topic of this manuscript falls within the scope of Nature Communications. I like the concept and material development/characterization proposed in this paper, moreover the manuscript includes in-depth studies to support the author claim from both fundamental and application point of views, anyway I think the manuscript needs a few improvements. I believe the article is of sufficient quality and novelty to meet the Nature Communications publication standard after a minor revision.

***Response:*** *We feel great thanks for your professional review work on our manuscript. The comments are very valuable to improve the quality of our manuscript. According to your sincere suggestions, we have checked our manuscript and revised it. We hope that you will find our responses satisfactory and the revised manuscript acceptable for publication.*

***Response to Reviewer 3***

**Comments to the Author:**

The manuscript by Zhang et al reports on the preparation of a polyacrylonitrile (PAN)-based composite membrane for sun-light driven dehumidification. Key of the finding reported here the combination of a bilayer system with a layer of PAN/carbon black layer for sunlight collection and another layer with PAN/metal organic framework (MOF)-LiCl nanoparticles for the conversion of gaseous water to liquid water. Once the liquid water has been collected the energy collected from sunlight will evaporate this water to outside and thereby dehumidify a room. Interesting the authors report on a special morphology of the electrospun membranes which they claim as wood-inspired morphology and is surely special for electrospun membranes. According to the authors this special morphology helps in dehumidification.

The manuscript is surely of interest for the readers of Nature Communication and could be also very inspiring for future work. The manuscript is well written and presents tons of experimental details. Only the figures are too small and the labels on many figures might be hard to read, which has to be improved.

***Response:*** *We truly appreciate your valuable comments and thoughtful suggestions. We have improved the pictures quality in the revised manuscript. We hope the corresponding changes to the paper will make it more acceptable for publication.*

Of course dehumidification by the MOF used here MIL-101(Cr) and the concept of using membranes for dehumidification is not new – please compare to doi: 10.1016/j.isci.2019.05.013; Membranes 2015, 5, 722-738; doi:10.3390/membranes5040722; <https://doi.org/10.1002/admi.201901310.> The authors should compare their systems with existing systems quantitatively and thereby elaborate the novelty of their system.

***Response:*** *We are very grateful for your valuable comment. Zhao et al. reported that* *an air dehumidification system was built which comprised nine pieces of one-inch PAN/poly(dimethylsiloxane) (PDMS) hollow fiber membrane modules^1^. The PAN/PDMS composite hollow fiber membranes were prepared using dry-jet wet spinning process and dip-coating method. The membrane modules worked under a low vacuum force of 0.78 bar absolute pressure at the lumen side. Air dehumidification by membrane separation technique is based on solution diffusion mechanism. Water molecules are preferentially permeating through the membranes due to its smaller kinetic diameter and higher condensability than the other gases. However, the experimental preparation and testing processes are complex and require high precision of the equipment.*

*Lunni et al. presented a hygroscopic bistable structure (HBS) inspired by the Venus flytrap leaves, obtained by bonding prestretched PDMS layers prior to depositing electrospun polyethylene oxide (PEO) nanofibers^2^. A hygroresponsive bilayer (HBL) was also obtained by electrospinning of PEO on an unstretched PDMS layer. HBS couples the hygromorphic capability of electrospun material with the mechanical advantage of preloaded structure to improve the actuation speed. As the carnivorous plant D. muscipula exploits a snap-buckling mechanism, the authors implemented a snap mechanism coupled with a hygroresponsive membrane to amplify the speed of closure. The developed plant-inspired soft bistable structure is used for sensing (e.g., humidity), which is fundamentally different from our manuscript in terms of material preparation, hygroscopic mechanism and application fields.*

*Cao et al. proposed a silica gel-MIL-101(Cr)-based moisture-permeable panel prepared using a partial immersion method, but the loading of MOF particles on the silica gel was unstable, and the particles easily agglomerated, which significantly reduced its water vapor transmission (WVT) and dehumidification capabilities^3^. To tackle this issue, it is highly desirable to develop self-supporting and flexible MOF nanofibrous membrane (NFM)-based desiccants with highly porous structures and a large number of active sites for moisture absorption and water harvesting. In this manuscript, we created a* *wood-inspired super hygroscopic moisture pump based on an electrospun NFM for solar-driven continuous indoor dehumidification. The manuscript reports the biomimetic bilayer NFM composed of a desiccant layer and a photothermal layer using a facile and scalable two-step electrospinning and impregnation method. Of particular interest is that electrospinning technology enables the assembly of MOF nanofibers into a highly ordered cellular network architecture, mimicking the wood structure. We highlight the following four innovative aspects of this work:*

(1) *Our approach, for the first time, enables the naturally fragile* *MIL-101(Cr) crystals to convert into flexible MIL-101(Cr) NFM with self-assembled 3D wood-like cellular networks and fluffy multilayered architecture using electrospinning technology directly. In this manuscript, we analyze in detail the 3D self-assembling process and mechanism of the multilayer wood-like cellular network structure.*
(2) *For the first time, an electrospun MOF NFM with super hygroscopicity, fast moisture absorption rate, and superior recyclability is prepared for moisture absorption and atmospheric water harvesting at high humidity. This is the first desiccant material based on flexible electrospun MOF NFM. The NFM-based desiccant exhibits an unprecedented moisture absorption capacity of 3.01 g g^-1^ at 25 °C and 90% relative humidity,* *and the moisture absorption capacity retains 99.4% of the initial capacity after 10 cycles, which is superior to those previously-reported desiccants. (0.45 g g^-1^ for Y-shp-MOF-5, J. Am. Chem. Soc. 2017, 139, 10715; 0.9 g g^-1^ for IPN gel, Nat. commun. 2018, 9, 2315; 1.04 g g^-1^ for PVA-LiCl NFM, Polym. Test. 2017, 64, 242; 1.58 g g^-1^ for MIL-101(Cr)@GO, Appl. Therm. Eng. 2015, 84, 118; 1.93 g g^-1^ for CaCl_2_@UiO-66 Adv. Funct. Mater. 2017, 27, 1606424; 2.3 g g^-1^ for hydrogel, Energ. Environ. Sci. 2018, 11, 2179.)*
(3) *Liquid water can be directly harvested from the humid air by absorbing moisture (gas) and oozing water using NFM-based desiccant without energy consumption. The concept of the phase transition of water molecules from the gas phase to the liquid phase will open up a variety of possibilities for developing flexible desiccants for energy exchange systems with low energy consumption.*
(4) *We propose a* *wood-inspired NFM-based moisture pump for solar-driven indoor dehumidification for the first time. This wood-inspired design offers distinct advantages for moisture pumping and water vapor exhaling. The moisture pump can efficiently reduce the indoor relative humidity to a medium level under one-sun illumination to meet human body’s comfort demands. Eventually, a moisture pump model using a moisture-permeable bilayer NFM is also designed. We analyze in detail the mechanisms of water absorption and water oozing for MOF NFM-based desiccant, and elaborate solar-driven continuous dehumidification mechanism of NFM-based moisture pump.*

*We envision that such a super hygroscopic NFM will result in new opportunities for developing atmospheric water harvesting, moisture-permeable windows on buildings, and humidity control inside electronic devices.*

Here, the inspiration by wood morphology is somewhat exaggerated. The explanation for the cellular-like structure of the membranes, which unique for electrospinning indeed, are assumptions and should be also identified accordingly (see page 10, top paragraph). Surely, this morphology was obtained accidentally and not planned. Therefore, one should not claim wood-inspired but better wood-like. In this context how it the scale of the wood morphology. Does it match the morphology of the present membranes? Further, is this morphology unique for composites with PAN/MOF or could it be obtained by other polymers or MOFs as well?

***Response:*** *Thank you very much for your valuable and important comments. In general,* *the fibers* *prepared by electrospinning were randomly oriented and continuous as a 3D open-cell nonwoven geometry; simultaneously, these fibers were highly interlaced with each other (Fig. R1). In our manuscript, according to SEM images (Fig. 2a-d), the micromorphologies of the prepared PAN/MIL NFM were analyzed in detail, and the structure forming process was modeled (Fig. 2e) by combining with the existing references (highlighted ref. 35-37). Through the establishment of the model, we have conducted a comprehensive analysis of the structure forming mechanism (see pages 9-10, lines 171-189).* *We think that the forming of the unique structure has certain theoretical references and our data support, and the model establishment and full explanation in our manuscript are enough to support the uniqueness of the structure forming process.*

**Fig. R1** SEM image of conventional PAN fibrous membrane prepared by electrospinning.

*As you mentioned, this morphology was indeed discovered by accident in an experiment, but then we carried out a series of regulation on the electrospinning conditions, and finally realized the controllable preparation of the biomimetic wood-like structure, and explained its forming mechanism. Obviously, “wood-inspired” refers to the fact that both the structure and properties of material are inspired by wood. However, “wood-inspired” in our manuscript is more concerned with the functionality of material.* *In other words, the design inspiration of hygroscopicity, moisture transportation and other properties of the moisture pump came from the properties of wood. This wood-inspired design offers distinct advantages for moisture pumping and vapor exhaling. Furthermore, the microstructures of the PAN/MIL NFM are similar to that of wood, which have cellular networks and interconnected open channels. Inspired by this special structures and unique functional characteristics, a biomimetic wood-like NFM was developed for moisture absorption and water vapor evaporation.* *Therefore, when describing the microstructures of NFM, we use the expression “wood-like” in the whole paper; when describing the properties of NFM or moisture pump, we use the expression “wood-inspired”. We have unified this expression in the revised manuscript.*

*As shown in Fig. 1c and d, according to statistical calculation, the average pore size of wood and PAN/MIL NFM was ~50 μm and 1 mm, respectively. The microscopic morphology of the two was similar, the scale of NFM morphology was larger. The large pores are conducive to moisture absorption, water vapor evaporation and water collection.*

*This morphology is unique to the conventional electrospun NFM, but is not specific to the electrospun PAN/MIL NFM. We have also tried electrospinning other polymers with MOFs, such as PEO/ZIF-8, PAN/HKUST-1. Fig. R2 presents the SEM images of the prepared PEO/ZIF-8 and PAN/HKUST-1 NFMs, revealing that the NFMs presented neatly-arranged cellular network structure and vertical open channels. Additionally, we speculate that other polymers such as PU, PCL, PLA, PVA, CA, etc. with MOFs can form similar cellular network structure by electrospinning, which will be further verified in future studies. Therefore, the preparation experiment is universal and scalable, and the prepared NFM will be applied to a wider range of fields according to the choice of polymer and MOF. In this manuscript, we have determined the polymer and MOF based on the application. We hope that you will find our responses satisfactory.*

***Fig. R2*** *SEM images of (a) PEO/ZIF-8 NFM and (b) PAN/HKUST-1 NFM.*

In Fig. 5 the authors suggest real applications which are of course of interest. Here, the question comes up about the UV stability of the sunlight-collecting layer – will it become brittle after some time? Has this been quantified?

***Response:*** *We truly appreciate your worthy comment and questions. To evaluate its practical applications, the prepared bilayer PML-PC NFM composed of a light green desiccant layer (PAN/MIL@LiCl NFM) and a black photothermal layer (PAN/CB NFM) was used as an NFM-based moisture pump. PAN/CB NFM was directly exposed to sunlight as a sunlight-collecting layer. After 130 min of sunlight irradiation, the bilayer PML-PC NFM still presented excellent flexibility and foldability (Fig. R3), which proved that PAN/CB NFM was not easy to become brittle after a period of exposure to sunlight, and it had good UV stability. On the one hand, from the molecular point of view, the -CN group in PAN can absorb UV light from sunlight and protect the molecular main chain. Therefore, the PAN nanofibers exhibit excellent UV stability^4,5^. On the other hand, CB can be used as an UV absorbent. When the CB aggregates dispersed in the PAN nanofibers are irradiated by UV light, they will cause UV scattering. The scattered light is mainly absorbed by the CB aggregates, so that the UV light absorbed by the polymer are reduced. Thus, the sunlight-collecting layer (PAN/CB NFM) in this manuscript showed excellent UV stability.*

***Fig. R3*** *Photograph of folding test of bilayer PML-PC NFM after dehumidification experiment.*

As a minor remark: why the photo in Fig. 3h was taken with different backgrounds?

***Response:*** *Thank you very much for your careful review.* *As shown in Fig. 3h, the stripped monolayer PAN/MIL@LiCl NFM demonstrated a more obvious hierarchical cellular network structure in the wet state. When the white building was taken as the background, the visual transmittance could be increased, so that the cellular networks composed of many three-branched fiber clusters could be seen more clearly. However, in the dry state, we focused on whether the liquid water on the NFM surface has completely evaporated and whether the color of the NFM has become lighter. Therefore, we used dark green plants as the background in the dry state, the changes of the NFM surface could be observed more clearly. The clear and enlarged optical photographs are shown in Fig. R4.*

***Fig. R4*** *Optical photographs of the stripped monolayer PAN/MIL@LiCl NFM after moisture absorption and drying.*

In conclusion, the manuscript by Zhang et al. is a very interesting manuscript and could be published in Nature Communication after minor revision.

***Response:*** *Thank you again for your comments on our manuscript. We have revised the manuscript according to the suggestions. We hope that you will find our responses satisfactory and convincing.*

**References**

1. Zhao, B., Peng, N., Liang, C., Yong, W. F. & Chung, T. Hollow fiber membrane dehumidification device for air conditioning system. *Membranes* **5**, 722-738 (2015).
2. Lunni, D., Cianchetti, M., Filippeschi, C., Sinibaldi, E. & Mazzolai B. Plant-inspired soft bistable structures based on hygroscopic electrospun nanofibers. *Adv. Mater. Interfaces* **7**, 1901310 (2020).
3. Cao, B., Tu, Y. & Wang, R. A moisture-penetrating humidity pump directly powered by one-sun illumination. *iScience* **15**, 502-513 (2019).
4. Ma L., Dong S., Shi J., Wang F., & Wang Y. Preparation of an anti-aging agent for polypropylene by waste polyacrylonitrile fabrics. *China Plast.* **3**, 82-89 (2017).

Ma L., Sun Z., He Z., Ji P., & Wang Y. Nano TiO_2_ grafted with polyacrylonitrile and its effects on anti UV aging properties of PP. *Eng. P*

**Reviewer comments, second version:**

Reviewer #1:
None

Reviewer #2:
Remarks to the Author:
The authors have responded well to comments,
Addressing my the criticisms I highlighted in the previous report Zhang et al. corrected all the technical inaccuracies present in the submitted manuscript, moreover they added numerous additional explanations, which will be very useful for the readers.
I believe the manuscript could be accepted for publication in the present form.


Reviewer #3:
Remarks to the Author:
The authors did some revision however I feel that they did not address the major points of of some serious critics. This mostly concerns from my point of view the attractive but misleading term wood-inspired and the sound discussion of the work of others in this direction in the manuscript. Therefore, in the present form I cannot recommend publication of the manuscript in a high ranking journal like Nature Communication.
I might add, that I am little surprised to see such a flat revision of the Ding group.

**Author rebuttal, second version:**

**Reviewer #2:**

**Comment**: The authors have responded well to comments.

Addressing my criticisms highlighted in the previous report, Zhang et al. corrected all the technical inaccuracies present in the submitted manuscript, moreover they added numerous additional explanations, which will be very useful for the readers.

I believe the manuscript could be accepted for publication in the present form.

***Response:*** *Thanks to the reviewer for the recognition of our work, and thank you for taking the time to review this manuscript. We are very pleased to hear that you recommended accepting our manuscript for publication in Nature Communications. The reviewer’s comments have greatly improved our manuscript.*

**Reviewer #3:**

**Comment**: The authors did some revision however I feel that they did not address the major points of some serious critics. This mostly concerns from my point of view the attractive but misleading term wood-inspired and the sound discussion of the work of others in this direction in the manuscript. Therefore, in the present form I cannot recommend publication of the manuscript in a high-ranking journal like Nature Communications.

I might add, that I am little surprised to see such a flat revision of the Ding group.

***Response:*** *We* *truly appreciate your comments and thank you for taking the time to review this manuscript. In your first comments, you clearly expressed your recognition of our work, and made valuable comments and thoughtful suggestions, which greatly improved our manuscript.* *In the previous revision, we really did our best to address your concerns point-by-point and revise the manuscript carefully based on your comments. Since you still have some concerns about the term “wood-inspired” and the comparison discussions with other works,* *here we make* ***a further explanation*** *about the term “wood-inspired”,* *and* ***provide discussions*** *in the manuscript.* ***For* *the term “wood-inspired”****, this wood-inspired design offers distinct advantages for moisture pumping and vapor exhaling. The microstructures of the PAN/MIL NFM are similar to that of wood, which have cellular networks and interconnected open channels. Therefore, when describing the microstructures of NFM, we use the expression “wood-like”; when describing the properties of NFM or moisture pump, we use the expression “wood-inspired” throughout the manuscript.* ***For the comparison discussions****, we add some comparison discussions in the introduction to further reflect the innovation of our research work (Please see page 5, lines 77-80). We hope that you will find our responses satisfactory.*

***Finally, we are sincerely thankful to the reviewers for the valuable comments.***

**Reviewer comments, third version:**

**Author rebuttal, third version:**
